# The rich phase structure of a mutator model

**DOI:** 10.1038/srep34840

**Published:** 2016-10-10

**Authors:** David B. Saakian, Tatiana Yakushkina, Chin-Kun Hu

**Affiliations:** 1 Institute of Physics, Academia Sinica, Nankang, Taipei 11529, Taiwan; 2 A.I. Alikhanyan National Science Laboratory (Yerevan Physics Institute) Foundation, Alikhanian Brothers St. 2, Yerevan 375036, Armenia; 3 National Research University Higher School of Economics, Moscow, 101000, Myasnitskaya Street 20, Russia; 4 National Center for Theoretical Sciences, National Tsing Hua University, Hsinchu 30013, Taiwan; 5 Business School, University of Shanghai for Science and Technology, Shanghai 200093, China

## Abstract

We propose a modification of the Crow-Kimura and Eigen models of biological molecular evolution to include a mutator gene that causes both an increase in the mutation rate and a change in the fitness landscape. This mutator effect relates to a wide range of biomedical problems. There are three possible phases: mutator phase, mixed phase and non-selective phase. We calculate the phase structure, the mean fitness and the fraction of the mutator allele in the population, which can be applied to describe cancer development and RNA viruses. We find that depending on the genome length, either the normal or the mutator allele dominates in the mixed phase. We analytically solve the model for a general fitness function. We conclude that the random fitness landscape is an appropriate choice for describing the observed mutator phenomenon in the case of a small fraction of mutators. It is shown that the increase in the mutation rates in the regular and the mutator parts of the genome should be set independently; only some combinations of these increases can push the complex biomedical system to the non-selective phase, potentially related to the eradication of tumors.

The concepts of genome instability^1^,^2^,^3^,^4^,^5^,^6^,^7^,^8^,^9^,^10^,^11^ and clonal evolutionary dynamics^12^,^13^,^14^ are key ideas in understanding the causes and behavior of cancer. Genome instability is a hallmark of cancer^11^, plays a significant role in bacterial evolution^15^,^16^,^17^,^18^,^19^, and has been a focus of physics-community research on oncology^20^. We need more complex nonlinear models^12^,^13^,^14^,^21^ than those for the viral evolution^22^,^23^,^24^,^25^ for the cancer analysis.

Models with relatively simple evolutionary dynamics can be applied to cancers that arise from one or two driver mutations, e.g., inherited retinoblastoma; however, most cancers require three or more. It has been proposed^1^,^26^ that some cancers may be associated with a substantial increase in the mutation rate of the genome. The question here is how the dynamics and phase structure of the evolving clonal population.

There have been some attempts to construct and solve evolutionary dynamics systems with mutator genes^16^,^17^. The earlier work^16^ on this subject, influenced by experimental results on *E. coli*^27^, discusses the problem of how selection in natural populations favors more rapid mutation. The authors suggest a simple model with a linear fitness function to identify the conditions that lead to selection for or against mutator states. Some approximate estimates were made for the steady state distribution and the dynamics of the mutator fraction in the population. The later paper^17^ used a quasispecies model with a multiplicative fitness function.

Classical quasispecies models^22^,^23^ describe the evolutionary process of an infinite population of binary sequences caused by two forces: mutation and selection; the former is controlled by the mutation rate and the latter by the fitness function. One of the most impressive results for these models is the existence of a phase transition^23^,^28^, known as the “error threshold” in some versions of the fitness landscape. When the mutation rate is small, a selective phase is observed, where sequences organize into a “quasispecies” near the sequence with the maximum fitness value. The non-selective phase observed with higher mutation rates shows dilution throughout the sequence space.

Returning to mutator dynamics, Nagar and Jain^17^ first studied the possible phases for these systems. Here, the fraction of mutator genome sequences in the population defines different phases: a normal phase with only normal sequences (a degenerate case when there are no back-mutations in a mutator gene), a mixed phase in which both mutators and non-mutators coexist, and a pure mutator phase with only mutators.

Among other findings, it has been shown that the small mutation rates for the mutator gene lead to the mixed phase with a small fraction of the mutator allele, but an increase in these rates turns the system to the mutator phase. The infinite population model with a constant mutation rate has been solved in ref. 29 (see also the recent review^30^).

When we map the evolutionary dynamics to statistical physics, the genome length plays the role of lattice size. In statistical physics, different phases arise as different analytical expressions for free energy at a large lattice size. Similarly, we need the large genome length to define the evolutionary dynamics phases. In this paper, we derive these different expressions for the mean fitness in mixed and mutator phases. The most realistic case, when back mutation for the mutator gene can be neglected due to its small impact during the time of the experiment, gives a very simple situation: in a mutator phase in a steady state, there is a zero fraction of normal sequences, even for small genome length, but there is a finite fraction of normal sequences in the mixed phase.

Several different classes of evolution models form the basis of mutator phenomenon analysis:

**Class I**     Phenomenological models^5^,^6^. Such models consider deterministic or stochastic equations for a set of several genotypes. Models in this class are relatively simple but useful for describing the specific types of experiments, e.g., investigations that show how the mutator mechanism competes with the ordinary evolution scheme with a high mutation rate^5^,^6^.

In ref. 5, a simple infinite population model without selection has been exposed, and it has been assumed that cancer arises after a given number of mutations in oncogenes. The probability of the start of cancer development at a fixed time has been calculated for the mutator mechanism as well as without mutators. The accuracy of the derivations has been well controlled using a small parameter: the ratio of the ordinary mutation rate to the mutation rate due to mutators. A different phenomenological model has been proposed without derivation details^6^, which discusses three types of fitness landscapes. This concept is of interest, and we consider one of the fitness landscapes in our work. However, the phenomenological models as a methodology cannot describe the different phases of the evolutionary dynamics accurately.

**Class II**     Infinite population microscopic models. Models in this class are defined in the binary sequence space with a finite genome length^16^,^17^. The Crow-Kimura^18^,^22^,^31^ and Eigen^23^,^24^,^25^,^32^ models, which are the quasispecies models mentioned above, have been widely investigated over recent decades, especially in the context of virus evolution. Mathematically, each of these models is described via a system of nonlinear master equations, which can be mapped to the chain of linear ordinary differential equations with some nonlinear transformation. In the Crow-Kimura setting, mutation and selection are parallel processes, while in the Eigen model, mutation is connected to selection. In smooth fitness landscapes, the steady-state distributions of the Crow-Kimura and Eigen models can be mapped to each other. In the large genome limit, the Crow-Kimura model is equivalent to a discrete-time Eigen model^33^ and is very close to branching processes. Moreover, the Wright-Fisher model^34^,^35^ for large populations can be mapped to a discrete-time Eigen model^33^.

**Class III**     Finite population microscopic models^36^. In this case, models are also defined in the binary sequence space^36^. In ref. 4, numerical simulations have been performed for the Moran model by considering the Muller ratchet in the presence of mutators. In ref. 7, the growth population version of the Wright-Fisher model^34^,^35^ for the four-dimensional landscape has been formulated; there are neutral, deleterious and advantageous mutations, as well as a sufficient number of mutations in the part of the genome that brings about the mutator effect. According to these results, the mutator phenotype evolves only at a moderate level of selection for the advantageous mutations.

**Class IV**     Infinite population and infinite number of genotypes models without backward mutations^29^,^30^. These models are useful to describe the virus evolutionary dynamics for a short period of time. Compared with the microscopic models, which represent different possible phases and collective effects in evolution, class IV models are less fundamental.

In the current paper, we consider the microscopic infinite population models (class II). The discrete-time version of the Eigen model^23^,^24^,^25^ was originally introduced to describe the self-replication of macromolecules at the origin of life (see also^37^,^38^). In this model, the selection and mutation happen together (mutation directly follows replication), while in the Crow-Kimura model, originally proposed to describe biological speciation, mutation and selection happen in parallel. In both models, the genome is represented by a chain of *L* letters (genes), which take the values ±1, similar to the Ising spin^39^,^40^. There is a mutation rate of *μ* per letter. We take the sequence with all “+” letters as the reference sequence. All of the genomes with the same total number *l* of “−” letters (*l* point mutations from the reference sequence) have fitness *r*_*l*_ ≡ *f*(1 − 2*l*/*L*), where *f*(*x*) as a fitness function, *x* = 1 − 2*l*/*L*. For the initial symmetric distribution, the model can be described via differential equations of *L* + 1 variables *P*_*l*_, corresponding to the total probabilities of the sequences with *l* mutations in the genome. The mean fitness of the Crow-Kimura model has been calculated using algebraic methods^41^ and the Hamilton-Jacobi equation (HJE) method^42^,^43^. The exact dynamics had been derived using the HJE^44^. Here, we construct a mutator gene model on the basis of the Crow-Kimura model^18^,^22^,^31^ and solve it for the general fitness case, by providing also the solution for the Eigen model version of the mutator phenomena.

Our solutions for both models are consistent with each other.

We find the analytical solution for the mean fitness for the general fitness function, including the multidimensional case, and clarify and calculate the order parameters and the phases of the model, which was an open problem until now. Previous work^5^,^6^ has been focused on the comparison between the efficiencies of cancer development via ordinary evolutionary dynamics and the evolutionary dynamics using the mutator mechanism. Our quantitative results of the phase structure (shown in Fig. 1) depict the borders of different phases. The procedure to obtain Fig. 1 is described below.

The phase structure may have biomedical implications. In ref. 9, it was proposed that the transition from the mixed phase to the non-selective phase is associated with the possible eradication of the tumor. We provide the solution for two types of fitness landscapes; a symmetric case, where the fitness landscape depends on the total number of mutations from the reference sequences, and a random fitness landscape.

Key technical terms and notations are listed in Tables 1 and 2, respectively.

## Results

### The model with a symmetric fitness landscape

In this section, we consider a modification of the Crow-Kimura model^18^,^22^,^31^ for evolutionary processes under the influence of a mutator gene. The chain of (*L* + 1) genes defines the genome, which can be formally subdivided into two parts; a regular part with *L* genes and a mutator gene, whose mutations change the fitness and the mutation rate in the regular part of the genome. We represent each gene with two alleles by *ξ*_*θ*_ = ±1, *θ* = 0, 1, …, *L*. The regular set of genes is denoted by the sequences *S*_*i*_ = (*ξ*_1_, …, *ξ*_*L*_), *i* = 1, …, 2^*L*^. The mutator gene in the state *ξ*_0_ = +1 determines the normal dynamics of the regular part with a mutation rate *μ*_1_. This wild-type form of the gene corresponds to the most common phenotype in the population. If the state of the mutator gene changes to *ξ*_0_ = −1, then the evolutionary process switches to a different regime with a mutation rate *μ*_2_. The mutant-type gene defines an altered phenotype, which is called a mutator phenotype. We use *α*_1_ and *α*_2_ to designate forward (*ξ*_0_: +1 → −1) and backward (*ξ*_0_: −1 → +1) mutation rates of the mutator gene.

We assume that the fitness landscape is symmetric, which means it depends solely on the number of mutations, *l*, from the reference sequence (without loss of generality, we take the reference sequence to be *S* = (+1, …, +1)). For this purpose, it is natural to use the notion of Hamming distance between *S* and 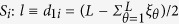 because all sequences in the same Hamming class have the same value of fitness. To facilitate further analysis, we introduce the variable for the mean value, 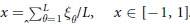. The state of the system is represented by two probability distributions. We use 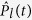 to denote the relative frequency of normal genome sequences (wild-types) with *l* mutations (in the *l*—th Hamming class) at the time moment, *t*, and 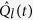 for the relative frequency of sequences with an increased mutation rate (mutator-types) with *l* mutations, and we have a probability balance condition for any time moment, 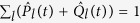. All admissible transitions between system states can be seen as arrows in Fig. 2 45,46.

To describe the evolution of the probability distribution under consideration, we use the following system of 2(*L* + 1) differential equations:


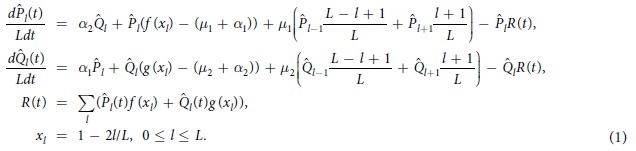


Here, *f*(*x*_*l*_) is a fitness function for the normal genome sequence, and *g*(*x*_*l*_) is a fitness function for the mutator sequence. As long as changes in the mutator gene significantly affect both the fitness and mutation rate in the regular part, fitness functions *f*(*x*_*l*_) and *g*(*x*_*l*_) can be different, and *μ*_2_ is commonly 10–100 times larger than *μ*_1_^2^,^10^. Note that the rates *α*_1_ and *μ*_1_ also differ because that *α*_1_ is proportional to the number of possible mutagenic loci. The coefficients (*L* − *l* + 1) and (*l* + 1) appear in the Eq. (1) transition terms according to the combinatorial formulae for Hamming class probabilities^41^,^47^. To investigate Eq.(1), we consider a nonlinear transformation^48^:


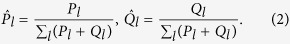


It is easy to show that *P*_*l*_ and *Q*_*l*_ are the solutions of the following system of linear equations:


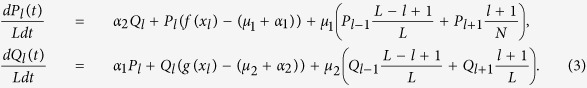


In Eqs (1) and (2), 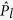 and 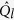 are the probabilities under the normalization constraint, while *P*_*l*_ and *Q*_*l*_ of Eq. (3) are not normalized.

In this paper, we focus on the following characteristics of the model in a steady state: the mean fitness *R*, the total surplus *s* (the expected value of *x*_*l*_), the surplus for the wild-type part of the population *s*_1_ and for the mutator-type part *s*_2_, and the fraction of the mutator sub-population *q*:


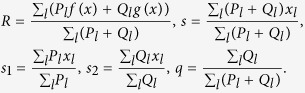


The surplus *s* shows the average number 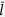 of mutations in the population, 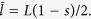 The maximum of the distributions *P*_*l*_ and *Q*_*l*_ is attained at points *L*(1 − *s*_1_)/2 and *L*(1 − *s*_2_)/2^44^. Therefore, the value of the mean fitness for wild-type sequences is equal to *f*(*s*_1_), and that for mutator-type sequences is equal to *g*(*s*_2_), as the distribution is narrow due to the large constant *L* in the exponent in Eq. (5) below. Assuming a smooth distribution, we obtain the following from Eqs (1) and (2):


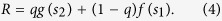


Calculating the mean fitness in growing populations is crucial for understanding evolutionary dynamics and is the primary concern of this investigation. Depending on the values of the main model parameters, we can obtain different analytical expressions for the mean fitness, which correspond to different phases of the system. The key problem is to first solve the large *L* limit, and clarify the following items:

- A. Is it possible to have selective and non-selective phases in the system with a phase transition between them?
- B. Does the average number of mutations depend on the state of the mutator gene or is it the same in both cases (*s*_1_ = *s*_2_)?
- C. Is it possible to have a significant fraction of normal genomes in the population: 1 − *q* > 0?
- D. Are there large finite *L* corrections for *q* at *L* ~ 10000?

These four questions are discussed in this paper; we show that both negative and positive answers exist for each. The second question (B) leads to the most considerable distinction in the analysis. In the Results section, we first investigate the case *s*_1_ = *s*_2_; then, in the Methods section, we describe a more complex situation with *s*_1_ ≠ *s*_2_. Moreover, contrary to the classical evolution models for quasispecies, here, the evolutionary picture depends on the genome length.

### The case of forward and backward mutations of the mutator gene

Let us consider a smooth solution of Eq. (3) at the limit *L* → ∞ after the simplification mentioned in the previous section. We assume the following ansatz^42^,^44^,^49^:


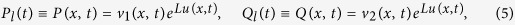


where we denoted *x* = 1 — 2*l*/*L* for large values of *L*. This form of expressions for *P*_*l*_ and *Q*_*l*_ allows the application of the HJE method to the system of equations (5) (see Methods). Putting our ansatz (5) into Eq. (3), we get a system of two equations for the variables *v*_1_, *v*_2_, *du*/*dx* and *du*/*dt*. Looking at these equations as a system of two linear algebraic equations for *v*_1_ and *v*_2_, we get a zero determinant condition for the non-zero solution of *v*_1_ and *v*_2_, so the final equation contains only *du*/*dt* and *du*/*dx*. The fact that *L* disappears in the Hamilton-Jacobi equation in the large *L* limit shows the correctness of our ansatz. We derive the mean fitness investigating the Hamiltonian without solving it and calculating the function *u*(*x*) in the steady state.

We obtain the following formulae for the mean fitness *R* using the potential function *V*_±_^44^:


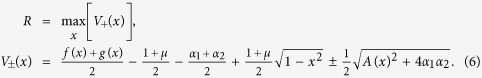


Here, we use *μ*_1_ = 1, *μ*_2_ = *μ*, and 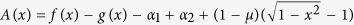. The numerical simulation for the system (1) supports the analytical result (6) well, as seen from Table 3.

Having the expression for the mean fitness *R*, we can calculate the surplus of distribution *s* from the following equation (see Methods):


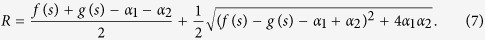


At the time limit *t* → ∞, we denote *v*_*k*_(*x*) ≡ *v*_*k*_(*x*, *t*), *k* = 1,2.

It is important to note that Eq. (7) is valid whether *s*_1_ = *s*_2_ or not. However, for *α*_1_ · *α*_2_ ≠ 0, we have that *s*_1_ = *s*_2_. In this case, substituting the solution of *R* and *s*, we get the following system of equations for the surplus *s* and ratio *v*_2_(*s*)/*v*_1_(*s*) in the steady state (see Methods) :


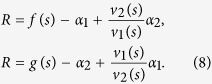


In the limit of large *t* and *L*, we have *q* = *v*_2_(*s*)/(*v*_1_(*s*) + *v*_2_(*s*)). For the case *f*(*x*) ≡ *g*(*x*), we get a simple relationship from (8):


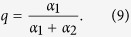


### The model with uni-directional mutations of the mutator gene

The probability of back mutation (from mutant to wild-type) is small and can be neglected in finite populations. Let us generalize this idea, and consider the infinite population model with *α*_2_ = 0, *α*_1_ = *a* (*μ*_1_ = 1, *μ*_2_ = *μ*). In this case, the expressions obtained for potential functions *V*_±_(*x*) are still valid, but it is necessary to take into account both branches of the potential, *V*_+_ and *V*_−_, and define the mean fitness as their maxima.

Taking *V*_−_(*x*) in Eq. (6), we get the mutator phase (here and below, we use terminology from^17^):


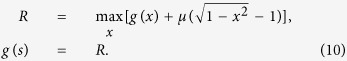


The latter equation for the surplus has the same form as for the ordinary Crow-Kimura model^41^. It can be derived directly from system (1) if we assume that the vast majority of the population has the mutator allele and omit the contribution of *P*_*l*_ in the second equation of Eq. (1). In the mutator phase, the mean fitness is defined by the fitness landscape for the mutator genome. Further, we will consider the case when two fitness landscapes are identical, e.g., *f*(*x*) = *g*(*x*).

Taking *V*_+_(*x*) in Eq. (6) under the condition *α*_2_ = 0, *α*_1_ = *a*, *μ*_1_ = 1, *μ*_2_ = *μ* leads to the following expression for the mean fitness of the mixed phase (see Methods):


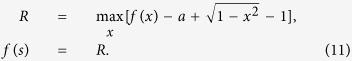


In the mixed phase, the mean fitness is defined by the wild-type fitness function and the mutation rate. However, numerical calculations for the linear fitness landscapes *f*(*x*) = *kx* or simple form quadratic fitness landscapes *f*(*x*) = *kx*^2^/2 show that the vast majority of the population has the mutator allele for a large enough genome length. It is worth pointing out that this result (the majority of population has a mutator-type) is correct for any smooth fitness landscapes for a long enough genome length. We verify that for the single peak fitness function, there is a finite fraction of wild-type sub-population at the large genome limit. Such results will be presented below.

Two phases have been found for the discrete-time Eigen model in ref. 17, where the mean fitness has been calculated for *f*(*x*) = *k*(*x* − 1), *k* ≫ 1. This model can be exactly mapped into the Crow-Kimura model at the large *L* limit, when the mutation rate per gene is fixed. Figure 3 gives a comparison of our analytical results for the mean fitness *R* with the numerics. Figure 3 shows that our analytical results are quite reliable.

### The single peak model with uni-directional mutations of the mutator gene

**Figure 1:**
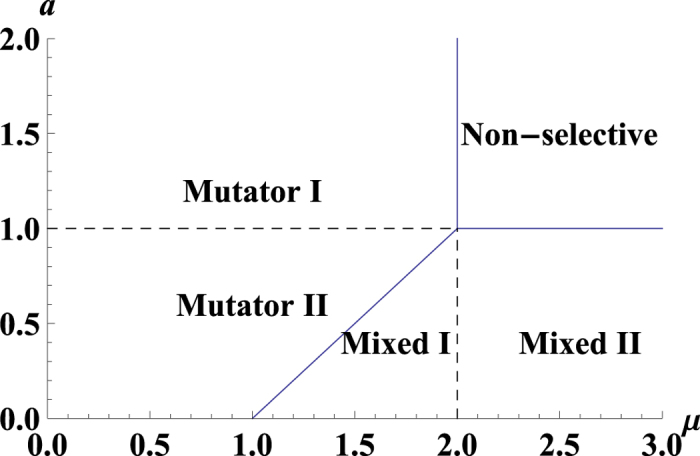
The phase structure of the mutator model with single peak landscape with zero value for any argument except *l* = 0: *f*(*x* = 1) = *g*(*x* = 1) = *J*. The parameters of the model are *α*_1_ = *a*, *α*_2_ = 0, *μ*_1_ = 1, and *μ*_2_ = *μ*. There are three phases: mixed phase with 0 < *s* < 1, 0 < *q* < 1, non-selective phase with *s* = 0, 0 < *q* ≤ 1 and mutator phase with 0 < *s*, *q* = 1. The border between non-selective and mutator phases is given by *μ* = *J*, the border between non-selective and mixed phases is given by *a* = *J* − 1, between mixed and mutator phases is given by *a* + 1 = *μ* line. From the bio-medical perspectives we distinguish the mutator I and mutator II, mixed I and mixed II subphases. From the mutator I, the system transforms to the non-selective phase simply increasing the *μ*. From the mixed II the system transformers to the non-selective phase simply increasing the *a* ≡ *α*_1_. From the mutator II and mixed I subphases we need change both a and *μ* to transform the system to the non-selective phase. We have the same picture in case of Eigen model with a fitness A for the peak sequence and fitness 1 for other sequences. We have for the mixed phase *R* = *e*^−(*h*+*γ*)^*A*, for the mutator phase *R* = *e*^−*μγ*^*A* and for the non-selective phase *R* = 1. The border between non-selective and mutator phases is given by *μγ* = ln*A*, the border between non-selective and mixed phases is given by *h* = ln*A* − *γ*, between mixed and mutator phases is given by *h* = (*μ* − 1)*γ* line.

**Figure 2:**
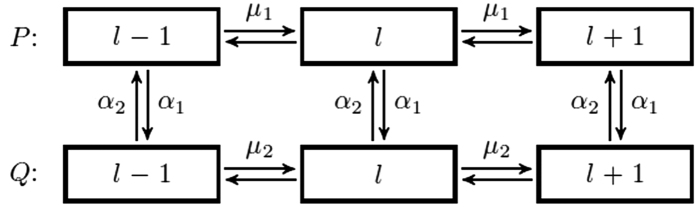
The scheme of available transitions for the system states (arrows denote transitions). The upper chain corresponds to the genome without a mutator allele (wild-type); the lower chain corresponds to the genome with a mutator allele (mutator-type). *l* is the number of mutations in the regular part of genome.

**Figure 3:**
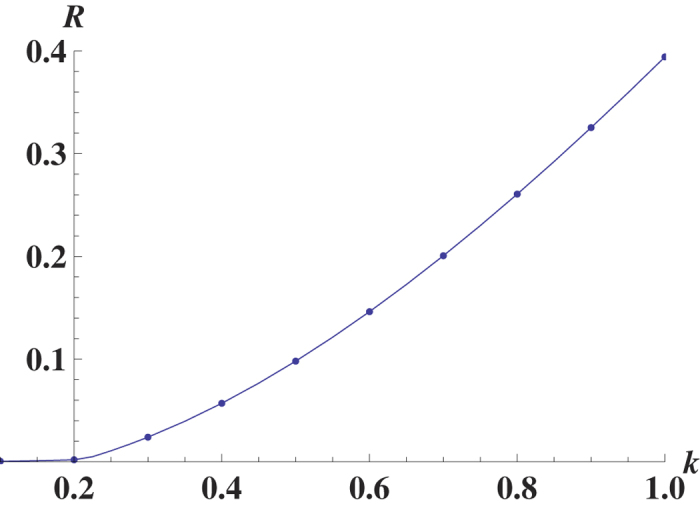
The mean fitness *R* versus *k* of the model with linear fitness landscape *f*(*x*) = *kx*, *μ*_1_ = 1, *μ*_2_ = 10, *α*_2_ = 0, *α*_1_ = 0.02. There are two phases in the model for the general values of parameters: mixed phase with 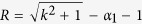 and mutator phase with 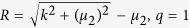. The border between two phases is given by equation 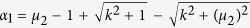. In our case *k*_*c*_ ≈ 0.212.

Let us consider a single peak fitness function, *f*(*x* = 1) = *g*(*x* = 1) = *J* and *f*(*x* ≠ 1) = *g*(*x* ≠ 1) = 0. We have three possible phases in a steady state (Fig. 1). We consider either the steady state of Eq. (1), or the asymptotic solution of Eq. (3), replacing 
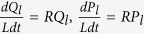
25. We provide the following expressions for the mean fitness in the mutator phase *R*_*mu*_, mixed phase *R*_*mix*_ and non-selective phase *R*_*ns*_; see Fig. 1:


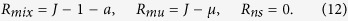


We first calculate *P*_0_ considering the equation for *dP*_0_/*dt* in Eq. (1) and ignore *P*_1_ term. The fraction of the wild-type sequences in the *l*-th Hamming class in the population is *P*_*l*_ = *P*_0_(1/*J*)^*l*^, which can be derived from Eq. (55) of ref. 50; therefore, for the fraction of the population with a normal mutator gene, we have 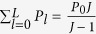. Considering analogously the equation for *dQ*_0_/*dt* and ignoring the *Q*_1_ term, we obtain 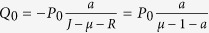. Conversely, the definition of *R* gives *P*_0_ + *Q*_0_ = *R*_*mix*_/*J* ≡ (*J* − *a* − 1)/*J*. Thus, we derive for *P*_0_ the following equation:


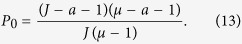


This expression allows us to calculate the mutator allele probability *q* using the equivalence 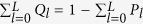:


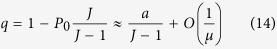


**Figure 4:**
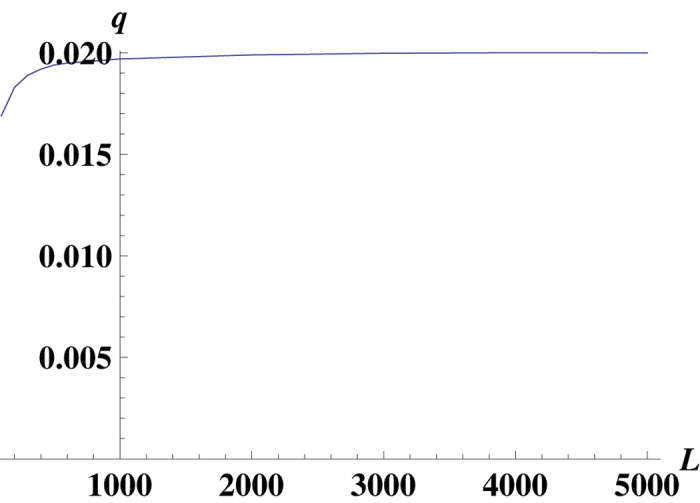
The dependence of the mutator probability *q* on the genome length *L*. The the single peak fitness model (smooth line) with *J* = 1.05, *μ*_1_ = 1, *μ*_2_ = 10, *a* = *α*_1_ = 0.001. For the *L* = 5000 the single peak model’s numerical result coincides with the analytical result for *L* = ∞ with the relative accuracy about 0.1%.

At *L* = 5000, the accuracy of our analytical result is approximately 0.1%, as seen in Fig. 4. Figure 4 and Eq. (14) show that the fraction of the wild allele does not approach to 0 even for a large *L*.

### The model with uni-directional mutations of the mutator gene and a smooth fitness landscape

As previously mentioned, we have the mixed and mutator phases. In the mutator phase, the numerics give that the mutator sub-population dominates with *q* = 1, even for finite *L*, so we can ignore the first chain of transitions and reduce the system to the standard Crow-Kimura model with mutation rate *μ*_2_ and fitness function *g*(*x*).

Let us now consider the mixed phase. If *α*_1_ = *a*, *α*_2_ → 0, then Eq. (8) gives *q* → 1 for this case. Assuming an ansatz 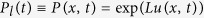, we obtain:


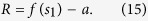


**Figure 5:**
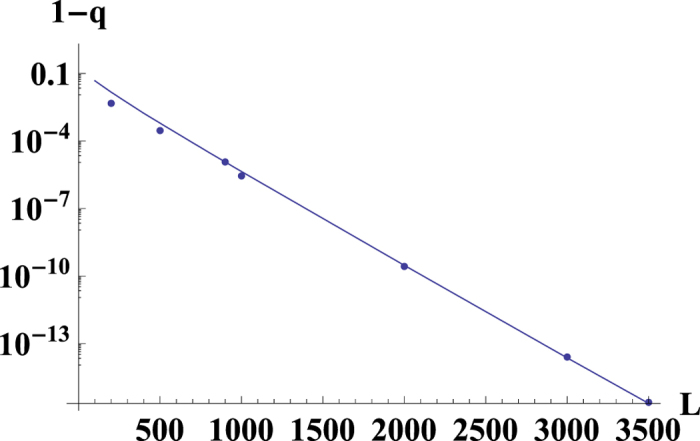
The dependence of the mutator probability *q* on the genome length *L* for the linear fitness model: *f*(*x*) = *kx*, *k* = 1, *μ*_1_ = 1, *μ*_2_ = 10, *α*_2_ = 0, *a* = *α*_1_ = 0.3. The smooth line corresponds to the numerics, the solid dots correspond to our analytical result.

**Figure 6:**
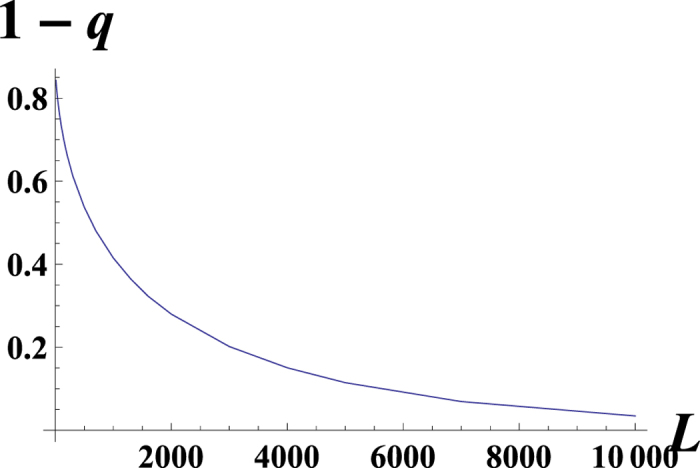
The dependence of the mutator probability *q* on the genome length *L* for 10 < L < 10000. We consider the linear fitness model: *f(x)* = *kx*, *k* = 1, *μ*_1_ = 1, *μ*_2_ = 10, *α*_2_ = 0, *a* = *α*_1_ = 0.05.

**Figure 7:**
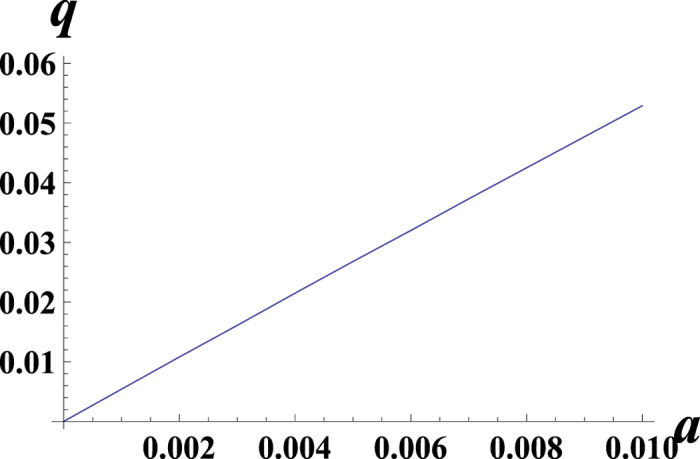
The dependence of the mutator probability *q* on the *a* = *α*_1_ for the linear fitness model: *f*(*x*) = *kx*, *k* = 1, *μ*_1_ = 1, *μ*_2_ = 10, *α*_2_ = 0, *L* = 1000.

The smooth curves in Figs 5 and 6 obtained by numerical calculations illustrate that the 1 − *q* strongly depends on the genome length, *L*. For the small genome length *L*, Fig. 7 obtained by numerical calculations support the behavior


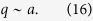


As has been derived in ref. 29, for the large *L*, we get 1 − *q* ≪ 1. In the Methods section, we calculate (1 − *q*) for the system with a general smooth symmetric fitness function and parameters *a*, *μ*. For linear fitness functions *f*(*x*) = *kx* and small values of *a* and *μ*_1_/*μ*_2_ ≡ 1/*μ*, we obtain a simple expression:


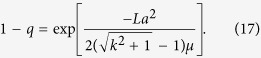


This result is well supported by the numerics, as seen in Fig. 5 and Table 4 for 1 − *q* ≪ 1, when the exponent is a large negative number. The transition between two regimes (16) and (17) is for the values of *L* where the expression of the exponent in Eq. (17) is ~1.

In population genetics, the fitness difference for one mutation is denoted by *S* and corresponds to the linear coefficient *k*; *U* is the total number of mutations per generation. We use the equalities *U* = *εμ*_1_, *S* = *ε*2*k*/*L* and *h* = *εα*_1_, where *ε* is the discrete time step of one generation and *h* is the transition probability to the mutator allele during one generation. Applying the discrete-time Eigen model to Crow-Kimura model mapping, we get a condition for the exponent:


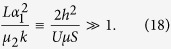


Otherwise, the numerics give *q* ≪ 1 instead of Eq. (17).

The linear fitness case can be solved exactly. We introduce the generation functions *Q*(*z*) = ∑_*l*_*Q*_*l*_*z*^*l*^ and *P*(*z*) = ∑_*l*_*P*_*l*_*z*^*l*^, so rewriting Eq. (1) with these variables gives a system of equations for the generation functions:


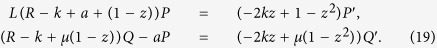


It is easy to calculate *R* from the first equation with the constraint that *P*(*z*) is an *L*-th order polynomial. Another constraint is the probability balance condition, *P*(1) + *Q*(1) = 1. Solving this system, we can calculate *P*_*l*_, *Q*_*l*_ with relative accuracy 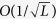. The generation function method has been applied before for investigation of the Crow-Kimura model^51^.

### The Eigen model with a mutator gene and random fitness landscape

The probability distribution for the Eigen model is defined for genome sequences with the wild and mutator type (*p*_*i*_ and *q*_*i*_, respectively, 0 ≤ *i* < 2^*L*^). Consider now the following system of equations


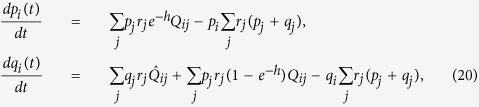


where *r*_*i*_ is the fitness function, *Q*_*ij*_ and 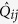 are probabilities of mutation from *S*_*j*_ to *S*_*i*_, *Q*_*ij*_ = *w*^*L*−*d*(*i*, *j*)^(1 − *w*)^*d*(*i*, *j*)^, *w* is the probability of errorless replication per nucleotide for sequences with normal mutator gene and 1 − *e*^−*h*^ ≈ *h* is the transition probability from the wild type to the mutator type. The diagonal terms of the mutation matrix are *Q*_*ii*_ = *w*^*L*^ ≡ *Q* ≡ *e*^−*γ*^, where *γ* = −*L*ln(*w*) ≈ *L*(1 − *w*) is the mutation parameter in the Eigen model, 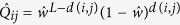, 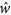 is the probability of errorless replication per nucleotide for sequences with a mutator allele. The mutation parameter for the mutator type is 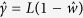. Here, *d*(*i*, *j*) is the Hamming distance between two sequences, *S*_*i*_ and *S*_*j*_.

For the single peak fitness function with *r*_0_ = *A* and *r*_*i*_ = 1, *i* ≥ 1 the mean fitness is similar to the one obtained for the Crow-Kimura model: *QAe*^−*h*^ for the mixed phase, 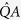 for the mutator phase and 1 for the non-selective phase. The random fitness landscape is a reasonable approximation for real biological data^52^. However, it was shown that it is almost equivalent to the single peak fitness landscape^33^. If we assume a log-normal distribution of Wrightian fitnesses, *r*_*i*_:


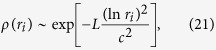


then, we have the maximal value of fitness 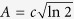
33, while for the majority of the population, fitness is equal to 1. Using the formulae for the single peak fitness case from the Methods section, we obtain:


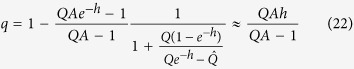


where we dropped the small term, *h*/*μ*. The steady state distribution is the same for the continuous-time Eigen model (20) and for the discrete-time Eigen model. For small values of *h* ≪ 1, *U* ≡ *γ* = *L*(1 − *q*) ≪ 1 and (*A* − 1) ≡ *S* ≪ 1, we get:


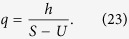


Equation (23) coincides with the Crow-Kimura model result in Eq. (14) after mapping *τJ* = *S*, *U* = *τ*, *h* = *aτ*, where *τ* is the growing time scale of the replicator, and we identified the Crow-Kimura model dynamics after the time *τ* with the dynamics of the discrete time Eigen model^33^.

It is interesting that there is no *μ* dependence in our expression. Taking the case of strong selection, *s* ≫ *U*, we obtain the situation when *q* ≪ *h*/*U*, as in the case of refs 53 and 54. According to ref. 53 (Table 1), we have *U* = 0.003, *q* = 0.00001 and *h*/*U* = 0.0001, while for ref. 54 (see abstract), we have *q* = 3/10^5^, *h* = 5/10^6^. The case of large *q* = 1/408 = 0.245%^55^, p. 109, perhaps is related to the smooth fitness landscape.

## Discussion

In this work, we have investigated the mutator phenomenon in the evolutionary process in a stable environment for infinite population size. Previous studies have obtained some results for a microscopic model with uni-directional mutation (wild-type → mutator-type) and linear Malthusian fitness in a 1-dimensional landscape. The mutator and mixed phases have been considered theoretically in the literature, so we continued to focus on the phase structure in this research. We have calculated exactly, for the first time, the phase structure of the model for the general fitness function, the analytical expression for the mean fitness for any scheme of mutations between normal and mutator states in the *d*-dimensional fitness landscape (see Methods). Moreover, we have discussed the important case of the random fitness landscape. We have derived the formula for the mutator-type probability, *q*, for the large genome length. This mathematical problem is highly non-trivial, as it is non-perturbative via the value of the back mutation rate: see Eqs (8) and (17). Equation (17) shows that the mutator-type takes over the entire population for the model with a linear fitness function. This effect has been observed experimentally^27^,^56^.

In ref. 17, an expression for the mutator allele probability has been derived when the mutator-type is the minority and the fitness function is linear. Our results confirm these findings^17^; furthermore, our method provides an analytical solution for the model in this case. When condition (18) is broken, the wild-type takes over the population, which has also been proven experimentally^54^. The value of *q* depends on the genome length (*q* ≪ 1 or 1 − *q* ≪ 1; see Fig. 5), and for the surplus, both alternatives hold (*s*_1_ = *s*_2_ or *s*_1_ ≠ *s*_2_). Thus, the finite size corrections could be large, even for the large genome length *L* ~ 10^4^.

A careful investigation of the evolutionary models is essential for biological applications. Looking at possible phases with order parameters is needed to provide an appropriate level of accuracy. As long as cancer is assumed to be a collective phenomenon, even with some collective intellect^57^, it is necessary to use analytical methods for complex systems. Let us compare our results with those by Desai and Fisher^29^ who solved a phenomenological model, while we rigorously solved the microscopic model. For “small” genome sizes, the mutator fraction is proportional to the small parameter *α*, as has been found in ref. 29. What we claim is *q* → 1 for sufficiently large genome length for any smooth fitness function. The latter case is a non-perturbative phenomenon, and it is not represented in the approach of^29^, as they neglected the backward mutation between different classes of wild-types. The approach by Desai and Fisher^29^ cannot be applied for the steady state of our model, as it is completely wrong for that case; however, Desai and Fisher obtained^29^ a useful (especially for the cancer case) analytical estimate for the short-time dynamics for the involved case of general fitness function.

Theoretical results concerning the error threshold^58^ have a practical impact for viruses and cancer, and it has been recently suggested to use an error catastrophe as a new therapeutic strategy for the solid cancer treatment^10^. A phase structure shown in Fig. 1 qualitatively describes the different stages of cancer; the mixed phase *s* ≠ 0 characterizes the early non-aggressive version of tumor, and the mutator phase is an aggressive stage of cancer, possibly with metastasis. To eradicate the tumor according to the strategy suggested in ref. 9, it should be transferred to the non-selective phase *s* = 0. As seen in Fig. 1, there are four different situations. In the mixed II subphase, we can push the tumor to the non-selective phase (the eventual goal of error-catastrophe therapy) to increase the forward mutation rate for the mutator gene *α*_1_, which is responsible for the genome stability. In the mutator I subphase, we should increase the mutation rate *μ* to push the tumor to the non-selective phase. In the mutator II and mixed I subphases, we need to increase both versions of mutation rates. The phase structure has the same form for the random fitness case and a similar form for other fitness landscapes.

Further research should focus on the finite population version of the mutator model, as the population size can drastically affect the evolutionary dynamics^8^. The problem has already attracted attention^19^, but it is reasonable first to solve a simpler case of infinite population limit.

To summarize, let us directly reply to the four questions stated in the beginning of the Results section.

- A. There is a phase transition between two phases, selective and non-selective, for the random fitness landscape and for the last two types of fitness landscapes considered in ref. 6 (the fitness landscape is flat until some critical number of mutations, then jumps and later is flat again; the fitness decreases linearly until some number of mutations and then jumps). For the linear fitness landscape, there is no phase transition.
- B. For small enough genome length *s*_1_ ≠ *s*_2_, see the Methods section for a detailed explanation.
- C. For a small genome length, it is possible to have some non-zero fraction of wild-types (see the Methods section).
- D. There are large finite *L* corrections for *q*; see Figs 5 and 6.

## Methods

To perform the analytical investigation Eq. (3), we use the HJE approach. Ignoring *O*(1/*L*) correction terms and using Eq. (5) and the formulae *P*_*l*±1_ = *v*_1_*e*^*Lu*±2*u*′^ and *Q*_*l*±1_ = *v*_2_*e*^*Lu*±2*u*′^ in Eq. (3), we get:


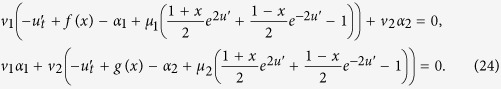


Here, we denoted 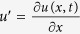 and 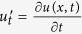. We consider Eq. (24) as a homogeneous system of equations for *v*_1_ and *v*_2_, so its determinant is equal to 0 for the non-zero solution of *v*_1_ and *v*_2_. First, we find 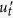 from the latter condition and write an equation, 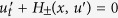, where *H*_±_ is a Hamiltonian. Then, we derive the potential function as


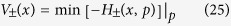


For the general case we have the following Hamiltonian:


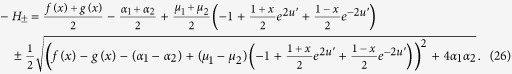


Deriving *V*_±_(*x*) according to Eqs (25) and (26), we get Eq. (6) to calculate the mean fitness *R*.

At the maximum point *x* = *s* of the distribution, we take 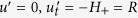 in Eq. (26) to obtain Eq. (7) for the surplus *s*. Putting to Eq. (24) the expression for the *R*, and looking the point *x* = *s*, we derive Eq. (8).

If f(x) = g(x), *α*_1_ = *a*, *α*_2_ = 0 (*μ*_1_ = 1, *μ*_2_ = *μ*), then, taking into account the ansatz *P*_*l*_ = *e*^*Lu*(*x*,*t*)^, we derive an equation:


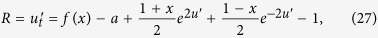


and the second equation for the mixed phase in Eq. (11): f(s) = R.

### The mutator-type probability

Consider the general case *s*_1_ ≠ *s*_2_ and parameters *α*_1_ = *a*, *α*_2_ = 0, *μ*_1_ = 1, *μ*_2_ = *μ* and *f*(*x*) = *g*(*x*). For *Q*_*l*_ ~ *P*_*l*_, we consider the second equation in (1) with the substitution *P*_*l*_ = *e*^*Lu*(*x*,*t*)^ in a steady state. We derive an equation for *Q*_*l*_:


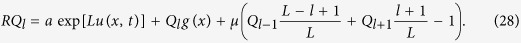


We can write an approximate solution:


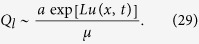


If *P*_*l*_ ≪ *Q*_*l*_, then, substituting 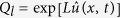, we get the following equation for 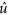:


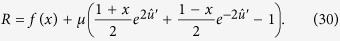


The expression for *s*_2_ can be derived from *R* = *f*(*s*_2_). We took the solution (27) at the interval [*s*_3_, 1] (where *s*_3_ is a switching point between *s*_1_ and *s*_2_) and the solution (30) at the interval [*s*_1_, *s*_3_]. We use the concatenation of these two solutions, assuming the smoothness of the derivative 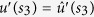, thus we obtain the following equation for *s*_3_:


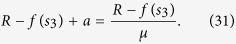


Putting a condition *u*(*s*_1_) = 0 (which is equivalent to ∑_*l*_*P*_*l*_ = 1) and 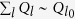, *l*_0_ = (1 + *s*_2_)*L*/2, we get:


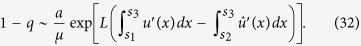


Here, *u*′ and 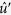 are calculated using Eqs (27) and (30), respectively. The expression in the exponent in Eq. (32) is exact at the limit *L* → ∞.

These analytical results allow us to predict, which allele takes over the population. The analysis of Table 4 reveals, that if 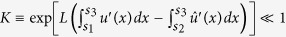, then the mutator-type dominates. If 1 − *K* ≪ 1, then the wild-type takes over the population.

### The case of small mutation rate for mutator allele *a*

Let us suppose that forward mutation rate *a* is sufficiently small. This assumption leads to the following equations:


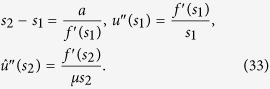


From (31), we obtain


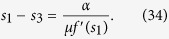


We are looking the case of large *μ*, therefore, we can replace *s*_3_ by *s*_1_:


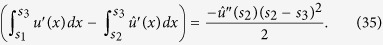


Thus


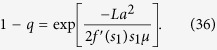


For the linear fitness *f*(*x*) = *kx*, we get Eq. (17):


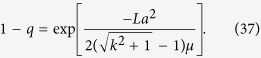


This solution is correct when the following condition is met:


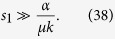


### The mean fitness for the model with *d*-dimensional fitness landscape and mutator gene

The multidimensional model has been discussed in ref. 19, where two parts of genome were considered; the first part is with lethal mutations and the second part is with deleterious and advantageous mutations. The genome is formally fractured into *d* parts with the lengths *Ly*_*i*_. For the wild-type, the fitness is equal to ∑_*i*_*f*(*x*_*i*_), and for the mutator-type, the fitness is ∑_*i*_*g*(*x*_*i*_), where *x*_*i*_ = 1−2*l*_*i*_/(*Ly*_*i*_) and *l*_*i*_ is the number of “−” spins (alleles) in the *i*-th part of genome. We denote mutation rates as *μ*_*i*_ for the wild-type and *ν*_*i*_ for the mutator-type, see ref. 49 for the multidimensional fitness model without a mutator gene. For these modified expressions, we calculate the mean fitness *R* as a maximum of the potential function *V*_±_, defined as:


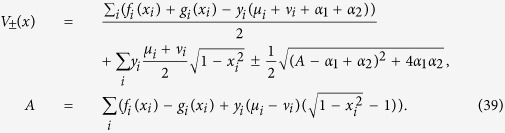


Here *α*_1_, *α*_2_ have the same meaning as in Eq. (1). The model considered in ref. 19 corresponds to the values: *d* = 2, *f*_1_(1) = *g*_1_(1) = 0, *f*_1_(*x*) = *g*_1_(*x*) = −∞ for −1 ≤ *x* < 1 and *f*_2_(*x*) = *g*_2_(*x*) = *k*_2_(1 − *x*), for the case of finite population.

### The Eigen setting of the mutator model

Consider the first equation in the system of Eq. (20) as an equation for the eigenvalue *R*. Compared with the ordinary Eigen model, the matrix of the linear system is multiplied by *e*^−*h*^. Thus, we can directly use the results from^58^ and write the expression for the mean fitness of the mixed phase for the fitness function *r*_*i*_ = *f*(*x*):


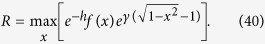


For the single peak fitness, we obtain for the selective phase^58^:


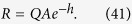


For the non-selective phase, we simply have *R* = 1.

In the mutator phase, we ignore the first chain of equations in Eq. (20) and get *R* as the mean fitness of the Eigen model with the value:


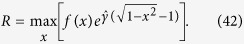


Consider now the distribution of population in the single peak fitness case. Ignoring the back mutations for the equation for *q*_0_ (because they give *O*(1/*L*) terms), we obtain:


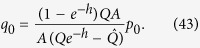


As all the sequences, besides *p*_0_, *q*_0_, have a fitness value 1, we have an equation (*q*_0_ + *p*_0_)*A* + (1 − *q*_0_ − *p*_0_) = *R*. Therefore


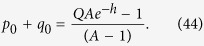


We derive eventually:


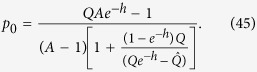


In the ordinary Eigen model


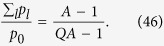


This equation is valid in our case as all the equations in the first chain of Eq. (20) are derived from the ordinary model^58^ by multiplying on *e*^−*h*^, moreover, we have that *Re*^*h*^ = *QA*. These two equations give together Eq. (22) of the text.

For the discrete-time Eigen model with a time step *n*, we have the probabilities *p*_*i*_(*n*), *q*_*i*_(*n*) and the following iteration equations:


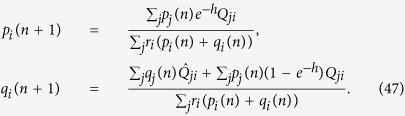


## Additional Information

**How to cite this article**: Saakian, D. B. *et al*. The rich phase structure of a mutator model. *Sci. Rep*. **6**, 34840; doi: 10.1038/srep34840 (2016).

## Figures and Tables

**Table 1:** Terms.

Lethal mutations	A mutation that results in the premature death of the organism carrying it.
Selective phase	A state of evolutionary system of binary sequences, when the majority of the population is concentrated near the peak genome (with the maximum fitness value) in the sequence space.
Non-selective phase	A state of evolutionary system of binary sequences, when the population is diluted in the sequence space.
Solvable cases of fitness landscape	Random fitness landscape and symmetric fitness landscape.
Random fitness landscape	The values of fitness defined on a sequence space are random numbers with some distribution.
Symmetric fitness landscape	The values of fitness defined on a sequence space by a function of the total mutation number.
Mutator phase	The absolute majority in the population has a mutator-type (mutator allele of the mutator gene) after long enough time of evolution.
Mixed phase	There is a finite fraction *q* of mutator sequences in the population after longtime evolution (0 < *q* < 1).

**Table 2:** Notations.

*L*	The genome length.
*l*	The number of mutations.
*ξ*_*θ*_, *θ* = 0,1, …, *L*	*ξ*_0_ = ±1 — the state of the mutator gene, *ξ*_*θ*_ = ±1 (1 ≤ *θ* ≤ *L*) — the states of genes in the regular part of the genome.
*S*_*i*_, *i* = 1, …, 2^*L*^	The genome sequence.
*r*_*l*_	The fitness value after *l* mutations in the Crow-Kimura model.
*P*_*l*_, *Q*_*l*_	The probability of the wild-types and mutator-types with *l* mutations.
*x*_*l*_ = 1 − 2*l*/*L*	The average gene state in the genome sequence that corresponds to *l* mutations from the reference sequence.
*f*(*x*_*l*_), *g*(*x*_*l*_)	The fitness functions of the wild-type and mutator-type genome sequences which assigns the reproductive rate to the average gene state *x*_*l*_
*μ*_1_, *μ*_2_	The mutation rate for the wild-types and mutator-types.
*μ*	The substitution used to denote briefly: *μ*_2_ = *μ* for the choice *μ* = 1.
*α*_1_, *α*_2_	The transition rates from the wild-type to mutator-type and vise versa.
*α*	The substitution used to denote briefly: *α* = *α*_1_ for the choice *α*_2_ = 0.
*s*	The surplus of the whole population: the average state of the gene.
*s*_1_, *s*_2_	The surpluses for the wild-types and mutator types.
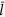	The average number of mutations, which is equal to (1 − *s*)*L*/2.
*q*	The total probability of the mutator-types.
*R*	The mean fitness.
*u*(*x*, *t*)	The probability of *l* mutations is 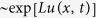 , Eq. (5).
*v*_1_(*x*, *t*), *v*_2_(*x*, *t*)	The relative probabilities of the wild and mutator types after *l* mutations, *x* = 1 — 2*l*/*L*, Eq. (5).
*V*_±_(*x*)	The potential of evolutionary dynamics, Eq. (26).
*k*	The coefficient in the linear fitness, *f*(*x*) = *kx*.
*J*	The peak fitness in the single peak fitness landscape for the Crow-Kimura model.
*U*	The total number of mutations for one generation in population genetics.
*h*	The transition probability wild-type to mutator-type for one generation in population genetics.
*ε*	The time period of one generation.
*p*_*i*_, *q*_*i*_	The probabilities of the *i*-th sequences for the wild type and mutator type in the Eigen model.
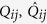	The transition probabilities between the sequences in wild-type and mutator-type in the Eigen model.
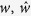	The probabilities of errorless replication per nucleotide for the wild-type and mutator-type.
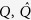	The probabilities of errorless replication for the genome the wild-type and mutator-type.
d(i, j)	The Hamming distance between the *i*-th and *j*-the genomes
*A*	The peak fitness in single peak fitness landscape for the Eigen model.
*τ*	The generation period of the virus.
*y*_*i*_	The relative length of the *i*-th piece of genome in multi-dimensional model.

**Table 3:** The comparison of the results for *f*(*x*) = *g*(*x*) = 3*x*^2^/2, *μ*_1_ = 1, *μ*_2_ = *μ*, *α*_1_ = *α*_2_ = 1, *L* = 400.

*μ*	3.5	3.0	2.5	2.0	1.5	1.0	0.5
*R*_*num*_	0.1907	0.2514	0.32405	0.4127	0.5240	0.6684	0.8626
*R*_*th*_	0.1811	0.2436	0.3180	0.4084	0.5212	0.6666	0.8615

*R*_*num*_ is the numerical result and *R*_*th*_ is given by Eq. (6).

**Table 4:** The results for *f*(*x*) = *g*(*x*) = *kx*, *μ*_1_ = 1, *μ*_2_ = *μ*, *α*_1_ = *a*, *α*_2_ = 0.

*L*	1000	1000	1000	1000
*k*	0.3	0.3	0.3	1
*a*	0.0001	0.001	0.01	0.3
*R*_*n*_	0.0439	0.0430	0.0340	0.1142
*R*	0.0439	0.0430	0.0340	0.1142
1 − *q*	0.9945	0.9460	0.530	6/10^7^
*K*	0.999996	0.9994	0.930	1/10^5^

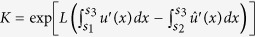
. *R*_*n*_ is the numerical result.
